# 

**Published:** 1875-07

**Authors:** 


### Table of Contents.

### ORIGINAL COMMUNICATIONS.

Seven Cases of Lithotomy—with comments on the history and treatment of calcareous affections. *By D. S. Brandon, M.D., Thomasville, Ga.*...... 183
Clinical Studies with Non-Nauseating Doses of Ipecacuanha, chiefly in Intermittents. *By Alfred A. Woodhull, M.D., Assistant Surgeon U. S. Army*...... 200
The Relations of Physicians and Druggists. *By Ferdinand King, M.D.* 214
Carbolic Acid as a Local Anæsthetic. *By Eugene Foster, M.D., of Augusta, Ga.*...... 219

### ATLANTA ACADEMY OF MEDICINE.

Spaying Sows—Peritoneal Wounds—Dysmenorrhœal Membrane, 221—Knot in Funis a cause of death of Fœtus—Uterine Polypus, 222—Ether vs. Chloroform, 223—Obstetric Anæsthesia, discussion, 225—Acute Peritoneal Cyst in boy, 228—Double Ovariotomy, case, 229—Dr. Campbell's Pneumatic Self-Repositor, discussion, 230—Amenorrhœa, case, 231—Ovariotomy, death, autopsy, remarks...... 232

### OUR EXCHANGES.

Doctors (poem)......
Newspaper Charlatans...... 237
Sponge Tents, preparation of......
Cancer Doctors...... 239
Fifteen Uses for the Indiarubber Condom...... 241
Opium Intoxication......
The Laborer is Worthy of his Hire...... 245
Traveling Hammocks for Invalids...... 246
Acute Dysentery, treatment...... 247

### BIBLIOGRAPHICAL.

Report of Hygiene, U. S. Army...... 247
Pamphlets...... 248

### EDITORIAL.

A Medical Legend...... 249
State Board of Health—the Law...... 250
Board of Health, Organization and Committees...... 255
Honors Accumulating......
Editorial Notices...... 256

